# The Relative Frequency of Oral Lichen Planus and Oral Lichenoid Lesions in a New Zealand Population Diagnosed at a Specialist Reference Centre

**DOI:** 10.1002/cre2.70330

**Published:** 2026-05-05

**Authors:** Ayesha Sameera, Muhammad Aiman bin Mohd Nizar, Haizal M. Hussaini, Paul Cooper, Alison M. Rich, Benedict Seo

**Affiliations:** ^1^ Faculty of Dentistry, Sir John Walsh Research Institute University of Otago Dunedin New Zealand; ^2^ Faculty of Dentistry Universiti Kebangsaan Malaysia Kuala Lumpur Malaysia

**Keywords:** clinical features, demographic profile, oral lichen planus, oral lichenoid lesions, retrospective study

## Abstract

**Objectives:**

There has not been a comprehensive study examining the epidemiology, clinical presentation, and histology of oral lichen planus (OLP) and oral lichenoid lesions (OLL) in New Zealand. The aim of this study was to determine the clinico‐pathologic manifestation in a New Zealand population.

**Methods:**

The study retrospectively analyzed OLP and OLL cases from the electronic database of the Oral Pathology Centre, University of Otago, that were clinically diagnosed and histologically confirmed. Their demographic, clinical, and histopathologic data were comparatively analyzed for statistical significance using Fisher's exact test and chi‐square.

**Results:**

Of all the samples received over the years 2011 to 2023, 232 were identified as OLP and 563 were identified as OLL. The patient's mean age was 45 years in OLP and 56 years in OLL, and the M:F ratio was 1:1.86 and 1:1.94, respectively. Buccal mucosa and gingiva were the most common sites involved in OLP, but in OLL, the tongue was the second most common site affected. For tongue presentation, OLP was associated with a higher frequency in the female sex, but no such sex predilection was seen in OLL, and these associations were statistically significant. The number of multiple intra‐oral anatomical sites affected was higher in the OLP groups compared to OLL.

**Conclusion:**

This is the first study to examine the relative frequency of OLP and OLL in a New Zealand population. The current data are consistent with global epidemiological data, with the exception that the female predominance was not seen for tongue OLL lesions. Accurately determining the relative frequency of OLP and OLL is crucial. This effort not only validates our current understanding of these specific lesions within the New Zealand population but also provides a robust, necessary baseline for all subsequent epidemiological and clinical studies.

## 1 Introduction

Oral lichen planus (OLP) is a chronic inflammatory disorder of unknown etiology with characteristic relapses and remissions, displaying white reticular lesions, accompanied or not by atrophic, ulcerative and/or plaque‐type areas. Lesions frequently involve the buccal mucosa and are bilaterally symmetrical. Desquamative gingivitis may be a feature (Warnakulasuriya et al. 2021; Muller et al. 2022). Meta‐analysis by Li et al. (2020) showed that the estimated global prevalence of OLP is 0.98%, being higher in women compared to men and specifically higher in ≥ 40‐year‐olds. That study also showed that Asian countries have a lower reported prevalence than non‐Asian countries, with the highest prevalence (6.04%) in Brazil and the lowest (0.02%) in India (Li et al. 2020). Notably, OLP has been shown to have a malignant transformation rate of between 1.14% and 1.37% (Giuliani et al. 2019; Fitzpatrick et al. 2014). OLP shares clinical and histological features with oral lichenoid lesions (OLL). The distinction is based primarily on clinical presentation and the presence of an identifiable etiological factor. OLLs are defined as oral lesions resembling lichen planus but lacking typical clinical features, meaning they may show asymmetry, unilateral distribution, or present as a reaction to dental restorations (contact lichenoid lesions) or are drug‐induced (drug‐induced lichenoid lesions) (Muller and Tilakaratne 2022). The oral manifestations of graft versus host disease can also mimic OLL but are directly related to a prior stem cell transplant and subsequent immunosuppression and should therefore be identified as a separate entity.

Histologically, both OLP and OLL typically present with basal cell liquefaction degeneration and a band‐like inflammatory infiltrate composed predominantly of T lymphocytes situated at the epithelial‐connective tissue interface. The clinical and histological diagnostic criteria required for determining OLP are described in detail by Cheng et al. (2016) and Warnakulasuriya et al. (2021) and are key for the differential diagnosis; shown in Table 1.

**Table 1:** Diagnostic criteria of oral lichen planus and oral lichenoid lesion based on previous proposals (Warnakulasuriya et al. 2021; Cheng et al. 2016).

	OLP	OLL
Clinical criteria	Presence of bilateral, more or less symmetrical white lesions affecting buccal mucosa, and/or tongue, and/or lip, and/or gingiva Presence of white papular lesions and a lace‐like network of slightly raised white lines (reticular, annular, or linear pattern) with or without erosions and ulcerations Sometimes presents as desquamative gingivitis	Unilateral or asymmetrical; bilateral, Linked to a trigger like a dental restorative material or a drug Presence of white papular lesions and a lace‐like network of slightly raised white lines (reticular, annular, or linear pattern) with or without erosions and ulcerations
Histopathologic criteria	Presence of a well‐defined band‐like predominantly lymphocytic infiltrate that is confined to the superficial part of the connective tissue Signs of vacuolar degeneration of the basal and/or suprabasal cell layers with keratinocyte apoptosis In the atrophic type, there is epithelial thinning and sometimes ulceration caused by failure of epithelial regeneration as a result of basal cell destruction. A mixed inflammatory infiltrate may be found	Presence of band‐like lymphocytic infiltrate, Often deeper, perivascular, or patchy infiltrate; may contain plasma cells/eosinophils. Basal cell lysis

Like OLP, OLLs are also classified as potentially malignant disorders, with some evidence suggesting their malignant transformation rate may be higher than that of OLP (Shen et al. 2012; Thornhill et al. 2003). Given the importance of correct classification for prognosis and management, and the current paucity of data on the comparative frequency of OLP and OLL in this region, the aim of the present study was to determine the relative frequency of OLP and OLL in a population from a single specialist reference centre for oral and maxillofacial pathology in New Zealand. Analyses were performed using standardized criteria for diagnosis over a 13‐year period. Clear and uniform distinction between OLP and OLL is required to facilitate identification of potential risk factors, addressing concerns regarding their potential to transform to oral cancer and provision of explicit therapeutic plans.

## 2 Methods

### 2.1 Data Collection

**Figure 1:**
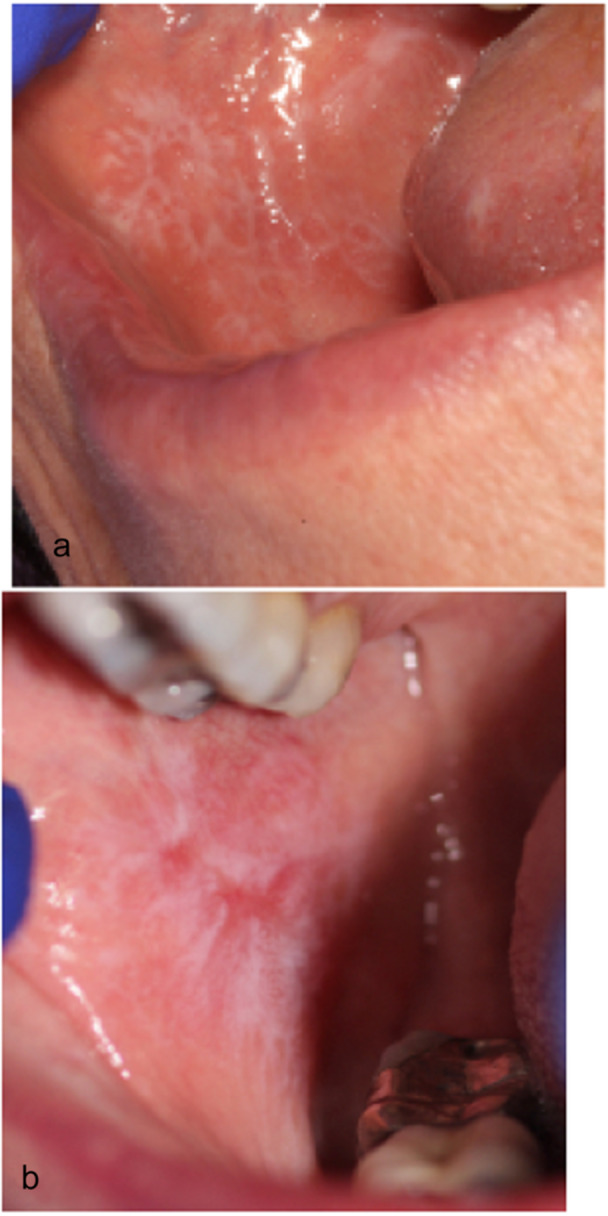
(a) Clinical presentation showing reticular type of oral lichen planus, and (b) oral lichenoid lesions in association with amalgam and cast metal restoration.

**Figure 2:**
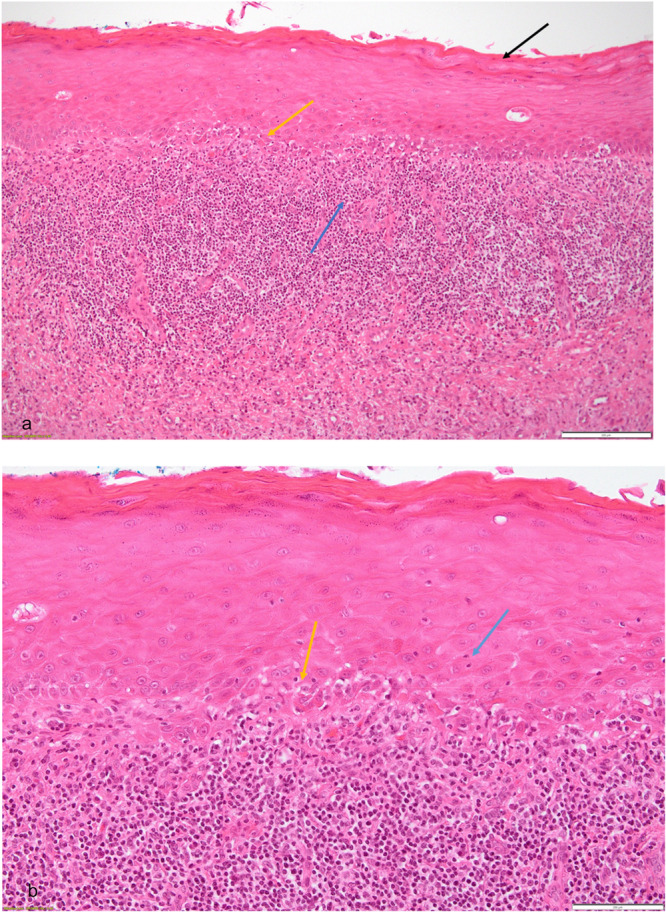
Histopathological features of oral lichen planus. (a) OLP lesion showing parakeratinised stratified squamous epithelium (black arrow) with basal cell degeneration (yellow arrow) and a continuous band‐like lymphocytic inflammatory infiltrate (blue arrow) at the epithelium‐connective tissue interface (H&E × 100). (b) Image showing basal cell degeneration (yellow arrow) with intraepithelial lymphocytic cell infiltration (blue arrow) (H&E × 200).

This was a comparative and retrospective study. The electronic database (years 2011–2023 inclusive) of the Oral Pathology Centre (OPC), Faculty of Dentistry, University of Otago, New Zealand, was searched for cases where a lesion, clinically diagnosed to be potentially OLP or OLL, was confirmed histologically (Figures 1 and 2). The OPC receives biopsies of oral and maxillofacial lesions from dentists, dental specialists, and anatomical pathologists from throughout New Zealand. The study was approved by the University of Otago Human Research Ethics Committee (H19/159).

The information provided by the referring clinician on the request form and from accompanying clinical photographs (when supplied) was extracted systematically by a single observer. This included patient demographical information (age and gender), information on the clinical presentation of the disease (such as site of distribution and clinical type), medical history, including current medication and information relating to tobacco smoking and alcohol consumption. In addition, pathological information was retrieved from the histopathology report. To ensure consistency across the 13‐year period and to minimize inter‐observer variability, all retrieved cases were reclassified and categorized as OLP or OLL based on the standardized criteria detailed in Table 1. The original diagnosis recorded by the reporting pathologist was not used for categorization, as the study's objective required consistent application of the chosen clinico‐pathological criteria across the entire cohort. Histology slides were only reviewed if the information in the report was incomplete or ambiguous; the study relied on the comprehensive diagnostic criteria applied during the case reclassification. All cases with epithelial dysplasia were excluded. After this initial survey, cases with incomplete clinical information or with inadequate or non‐representative histological sections were excluded from the final sample analysis.

A single anatomical site of involvement was defined as the presence of OLP or OLL in a single intraoral location (e.g., buccal mucosa, irrespective of the right or left side, is considered a single anatomical site) and multiple anatomical sites of involvement were defined as the presence of OLP or OLL in two or more distinct intraoral sites (e.g., buccal mucosa, tongue or gingiva).

### 2.2 Statistical Analysis

Fisher's exact test and chi‐square test were used to determine whether there were any statistically significant differences in the demographic and clinical profile of OLP and OLL in patients. The Student's *t*‐test was used to compare the mean age at diagnosis between the OLP and OLL groups. A *p* value < 0.05 was considered statistically significant. All statistical analyses were conducted using GraphPad Prism (version 10.4.1 (627), GraphPad Software, Massachusetts, USA, www.graphpad.com).

## 3 Results

In total, 802 cases were identified in the retrospective survey of the electronic database. Of those, seven cases with incomplete clinical and/or histological information were excluded, resulting in the final sample size of 795 cases. Of the 795 cases, 232 met the histological and clinical criteria for the diagnosis of OLP (29.2%), and 563 (70.8%) were classified as OLL.

**Figure 3:**
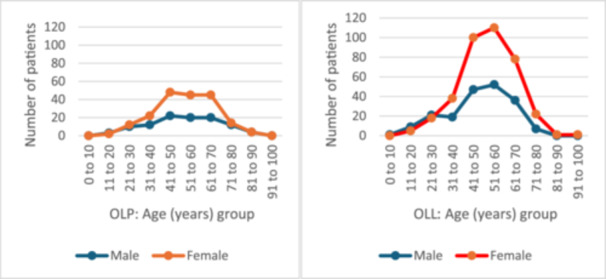
Age distribution of patients in oral lichen planus (OLP) and oral lichenoid lesions (OLL).

Sixty‐five percent of the OLP cases involved female patients (*n* = 151) and 81 were from male patients (34.9%). The mean age at diagnosis was 45 years, both in males and females (range 20–80 years) (Figure 3). Buccal mucosa was the most commonly affected site for both females and males; gingival and tongue involvement was reported in 21% and 16% of females (*n* = 49 and 38) as opposed to 13% and 6% of males (*n* = 30 and 14), respectively (Table 2).

**Table 2:** Demographic and clinical profile of the study.

Criterion	OLP group (*n* = 232, 29.1%)	OLL group (*n* = 563, 70.8%)	*p*‐value (< 0.05)
*Gender*			
Female	151 (65.08%)	372 (66%)	0.789
Male	81 (34.91%)	191 (33.9%)	
Female: Male ratio	1.86:1	1.94:1	
*Age*			
Mean age	45 years	56 years	
Age range	20–80 years	10–100 years	
*Clinical site*			
Buccal mucosa	162 (69.8%)	349 (61.9%)	0.916
Female	106 (45.68%)	230 (40.8%)	
Male	56 (24.13)	119 (21.13%)	
Gingiva	79 (34.05%)	125 (22.20%)	0.172
Female	49 (21.12%)	89 (15.8%)	
Male	30 (12.9%)	36 (6.39%)	
Tongue	52 (22.41%)	130 (23.09%)	0.027
Female	38 (16.37%)	72 (12.78%)	
Male	14 (6.03%)	58 (10.30%)	
Labial mucosa	6 (2.5%)	15 (6.46%)	0.305
Female	5 (2.15%)	9 (1.59%)	
Male	1 (0.43%)	6 (1.06%)	
Palate (soft/hard)	4 (1.72%)	5 (0.88%)	0.293
Female	1 (0.43%)	3 (0.53%)	
Male	3 (1.29%)	2 (0.355%)	
Floor of the mouth	2 (0.86%)	5 (0.88%)	0.850
Female	2 (0.86%)	3 (0.53%)	
Male	0	2 (0.355%)	
Retromolar area	1 (0.43%)	15 (6.46%)	0.787
Female	0	9 (1.59%)	
Male	1 (0.43%)	6 (1.06%)	
Pterygomandibular raphe	0	1 (0.177%)^a^	
Female			
Male			
Palatoglossal arch	0	1 (0.177%)^a^	
Female			
Male			
*Clinical type*			
Reticular	126 (54.3%)	194 (34.45%)	0.063
Plaque‐like	84 (36.2%)	175 (31%)	
Erosive	26 (11.2%)	74 (13.14%)	
Atrophic	47 (20.25%)	79 (14.03%)	
*Number of anatomical sites involved*			
Single	157 (67%)	408 (72%)	0.175
Multiple (≥ 2)	75 (32.32%)	155 (27.53%)	

*Note: p*‐value was calculated using the chi‐squared test, *p*‐value less than 0.05 was used in the rejection of the null hypothesis.

^a^ Sample size too small for statistical analysis.

**Figure 4:**
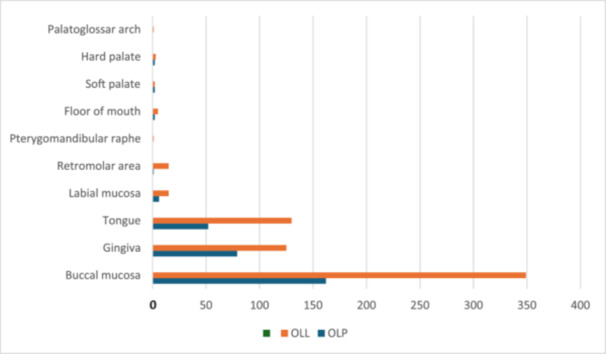
Site of involvement amongst patients with oral lichen planus (OLP) and oral lichenoid lesions (OLL).

Of the OLL cases, 66% came from female patients (*n* = 372) and 34% from male patients (*n* = 191) (Table 2). The mean age at diagnosis was 56 years, both in males and females (range 10–100 years). Buccal mucosa was the most frequently affected site for both females and males, followed by gingiva, with 15.8% and 6.4% in females and males, respectively. Tongue involvement was 12.8% in females and 10.3% in males (Figure 4). More females were represented in the current data set (female: male ratio of 1.86:1 and 1.94:1 in OLP and OLL, respectively) (Table 2; Figure 3).

Multiple anatomical sites were more likely to be involved in OLP groups (32.3%) in comparison to OLL (27.5%), with a single anatomical site involved in 67% and 72% in OLP and OLL groups, respectively; however, this did not reach statistical significance (*p* < 0.196).

**Figure 5:**
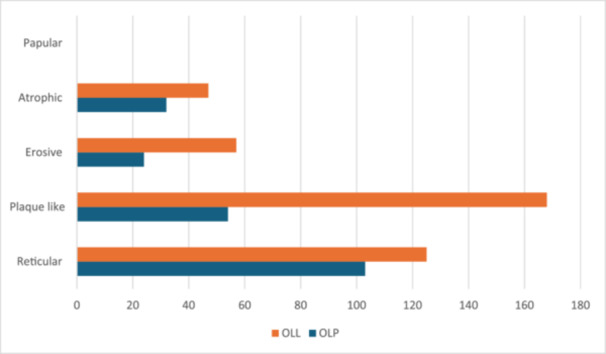
Clinical types distribution among patients with oral lichen planus (OLP) and oral lichenoid lesions (OLL).

Regarding clinical presentation (Figure 5), in the OLP group the reticular form was the most common (126 cases, 54.3%), followed by plaque‐like (84 cases, 36.2%); erosive (26 cases, 11.2%) and atrophic cases were 47 (20.25%) and in the OLL group the reticular form was the most common (194 cases, 34.45%), followed by plaque‐like (175 cases, 31%); erosive with 74 cases (13.14%) and atrophic cases were 79 (14.03%). The bullous form was not present in either of the conditions.

Regarding the histological features (Table 3), the presence and distribution of parakeratosis and orthokeratosis were observed similarly in OLP and OLL cases, (59.9% and 11%) and (54.4% and 8.1%), respectively. Epithelial atrophy was seen more in the OLLs (32 patients, 13.8%) compared with OLP cases (122 cases, 21.6%). Acanthosis was more common in OLP cases compared to OLLs (36 patients, 15.5% and 63 patients, 11.15%, respectively). Subepithelial clefting was found in 20 (8.6%) and 30 (5.3%) patients in the OLP and OLLs groups, respectively. All patient samples diagnosed as OLP showed basal cell lysis and a band‐like predominantly lymphocytic inflammatory infiltrate at the epithelium‐lamina propria interface. Similarly, all OLL cases exhibited basal cell lysis and had a predominantly lymphocytic inflammatory infiltrate, of which 93.4% (526 patients) had a band‐like pattern, and significantly, 6.4% of OLL cases exhibited a non‐band‐like inflammatory pattern (focal, diffuse, or patchy), compared to 0% of OLP cases (*p* < 0.001). The presence of macrophages in the immune infiltrate was noted in 30.6% (71 cases) OLP and 26.2% (148 cases) of OLL.

**Table 3:** Histological features distribution in OLP and OLL.

Histopathological features	OLP group	OLL group
*Keratinization*		
Parakeratosis	139 (59.9%)	306 (54.35%)
Orthokeratosis	19 (8.1%)	62 (11%)
*Atrophy*	32 (13.79%)	122 (21.6%)
*Acanthosis*	36 (15.5%)	63 (11.1%)
*Civatte bodies*	83 (35.7%)	168 (29.8%)
*Eosinophilic coagulum*	7 (3%)	31 (5.5%)
*Saw tooth rete pegs*	3 (1.2%)	7 (1.2%)
*Eosinophils*	7 (3%)	7 (1.2%)
*Plasma cells*	4 (1.7%)	11 (1.9%)
*Macrophages*	71 (30.6%)	148 (26.2%)
*Mast cells*	1 (0.4%)	1 (0.17%)
*Inflammatory infiltrate*	Band like‐232 (100%)	Band like‐526 (93.4%)
*pattern in connective tissue*		Focal‐6 (1%)
		Diffuse‐14 (2.4%)
		Patchy‐17 (3%)
*Subepithelial clefting*	20 (8.6%)	30 (5.3%)
*Melanin incontinence*	3 (1.29%)	9 (1.59%)

## 4 Discussion

The present study selected cases and categorized them as OLP or OLL according to strict criteria described above. The published data available on prevalence and comparison of OLP and OLL have been derived from Croatia, India, Brazil, Spain, Switzerland, Iran, Italy, and Tunisia (Mravak‐Stipetic et al. 2014; Netto et al. 2022; Juneja et al. 2006; Aguirre‐Urizar et al. 2020; Feldmeyer et al. 2020; Aminzadeh et al. 2013; Boñar‐Alvarez et al. 2019; Belkacem Chebil et al. 2019). We provide the first study on the relative frequency of OLP and OLL in a New Zealand population using standardized clinico‐pathologic criteria.

The Oral Pathology Centre is the primary national specialist reference centre for oral and maxillofacial pathology in New Zealand. As biopsies are received from dentists, specialists, and pathologists across all major geographical regions of the country, the sample cohort is likely representative of the spectrum of OLP and OLL cases referred for specialist histopathological assessment within the New Zealand healthcare system (Rich et al. 2007; Seo et al. 2017).

In our study, more females (nearly 65%) were affected by both OLP and OLL than males, and this is consistent with previous reports (Netto et al. 2022; Aguirre‐Urizar et al. 2020; Feldmeyer et al. 2020; Aminzadeh et al. 2013; de Lima et al. 2019). Comparable to the present study, which identified a mean age of diagnosis as 45 years for OLP and 56 years for OLL, other studies also reported that most patients were diagnosed with OLP in the fourth to sixth decade of life, with a mean age ranging from 45 to 65 years (de Lima et al. 2019; Pakfetrat et al. 2009; Shen et al. 2012). The finding of a significantly older mean age at diagnosis for OLL (56 years) compared to OLP (45 years) is consistent with the general pattern seen in other cohorts, such as reported by Issa et al. (2004) (OLL mean age 61 years) and Netto et al. (2022) (OLL mean age 57.9 years), suggesting that the etiological factors often associated with OLL (e.g., drug reactions or old restorations) become more prevalent with age (Mravak‐Stipetic et al. 2014; Lodolo et al. 2022).

For OLP, following the buccal mucosa, the gingiva was the most commonly affected site, followed by the tongue. This is in contrast to OLL, where the tongue was the second most commonly affected site, as has been previously reported in literature (Aminzadeh et al. 2013). Involvement of the tongue in OLL was seen with equal frequency in females and males; however, in OLP, tongue involvement was more prevalent in females (*p* < 0.03). This difference in the anatomical site predilection (higher tongue involvement in OLL) may reflect the known higher incidence of contact lichenoid lesions on the tongue, which would contribute to the OLL classification. In the present study, multiple anatomical sites were involved in approximately a third of all the cases of OLP and OLL groups. The findings are in keeping with previous studies (Netto et al. 2022; Juneja et al. 2006).

It is not uncommon for patients to present with both hyperplastic keratotic forms (reticular, papular, plaque‐like) and atrophic forms (erosive, ulcerated) simultaneously (Aguirre‐Urizar et al. 2020). In the current study, the reticular pattern was the most common disease presentation in both OLP and OLL, which corresponds with previous data (Juneja et al. 2006; Feldmeyer et al. 2020; Lodolo et al. 2022). However, when examining the groups separately, the reticular form was clearly more prevalent within the OLP group (54.3%) than within the OLL group (34.45%). This difference is important as it suggests OLL may encompass a broader range of clinical morphologies, consistent with its more diverse etiology.

Accurate distinction between OLP and OLL has important implications for clinical management strategies and in determining the prognosis of the diseases. Having a clear spatial and temporal association of a lesion with dental materials, such as an amalgam restoration, is often sufficient to justify a diagnosis of a lichenoid reaction, and in such cases, replacement of the restoration usually results in resolution in a large proportion of cases (Thornhill et al. 2003). Similarly, lichenoid drug reactions can be eliminated by changing medication regimes (Cawson 2002). Having clear criteria for separating OLP from OLL facilitates consistent treatment for both types of lesions.

Histologically, while both conditions share key features, there are slight differences in the infiltrate. In this study, 100% of OLP cases exhibited the classical band‐like pattern, while a small but significant fraction (6.4%) of OLL cases showed focal, diffuse, or patchy infiltrates. Additionally, OLL has been reported to often show a deeper inflammatory infiltrate, a higher presence of plasma cells, or an eosinophilic coagulum (Civatte body), which are less characteristic of classical OLP (Cheng et al. 2016).

Additionally, there has been considerable discussion concerning the malignant potential of OLPs and OLLs; both conditions have been identified as potentially malignant disorders (Warnakulasuriya et al. 2021) with increasing evidence that the transformation rate of OLLs may be greater than that of OLPs (Lodolo et al. 2022; González‐Moles et al. 2019). The most common subsite for malignant transformation was the tongue (Giuliani et al. 2019; Fitzpatrick et al. 2014). In our current study, the tongue was the second most commonly affected in OLL. Clinicians need to be aware that an OLL involving the tongue needs particular care and thorough follow‐up. This is another important reason to differentiate between the two diseases to ensure that treatment can be more targeted and specific.

However, the proposition that OLL carries a higher malignant risk than OLP is controversial and may be linked to methodological issues in previous studies. Some cohorts, such as those cited by Warnakulasuriya et al. (2021) and Gonzalez‐Moles et al. (2019), did not rigorously exclude cases with co‐existing epithelial dysplasia. Studies by Shearston et al. (2019) and Idrees et al. (2021) suggest that when true epithelial dysplasia is systematically excluded, the transformation risk for OLP and OLL is significantly lower or possibly comparable. Since all cases with epithelial dysplasia were excluded from our study, this potentially confounding factor was minimized, strengthening the validity of our distinction between the two entities.

Most cases of OLP are relatively mild, and symptoms can be controlled by intermittent topical corticosteroids (Idrees et al. 2021), but alternative therapies are necessary for recalcitrant atrophic and/or ulcerative lesions, which are painful and limit quality of life (Didona et al. 2022). This may involve intralesional or systemic corticosteroids, with or without the use of various corticosteroid‐sparing agents. Calcineurin inhibitors have shown some promise, but they are not always shown to be more effective than steroids (Ujiie et al. 2022). There have been reports of the effectiveness of anti‐interleukin monoclonal antibodies in the management of refractory OLP and the call for further research into the potential for this form of treatment (Ujiie et al. 2022; Al‐Hashimi et al. 2007). The key to the management of OLL is to identify the initiating factor. Treatment with topical or systemic agents without withdrawal of the initiator will not be effective. Consequently, understanding the occurrence of OLP and OLL in New Zealand and correctly separating these lesions into their respective categories will aid in the development of consistent management protocols, which in turn will enhance the validity of future clinical and research studies.

## Author Contributions

**Ayesha Sameera:** conceptualization, methodology, validation, formal analysis, writing – original draft, writing – review and editing. **Aiman Mohd Nizar:** data curation, formal analysis. **Haizal Mohd Hussaini:** formal analysis, data curation, and editing. **Paul Cooper:** supervision and critical review. **Alison Rich:** writing – review and editing, supervision. **Benedict Seo:** formal analysis, validation, review and editing.

## Funding

The authors have nothing to report.

## Conflicts of Interest

The authors declare no conflicts of interest.

## Data Availability

The datasets analyzed during this retrospective study on oral lichen planus and oral lichenoid lesions in New Zealand are not publicly available to protect patient confidentiality. Access to the raw data, which contains sensitive personal information and clinical photographs, is restricted in accordance with the ethical approval guidelines of the Sir John Walsh Research Centre, University of Otago, Dunedin, New Zealand.
